# Fibronectin as a Marker of Myocardial Remodeling in Patients with Depression and Chronic Heart Failure

**DOI:** 10.1192/j.eurpsy.2024.760

**Published:** 2024-08-27

**Authors:** A. K. Sikora, S. Fedorov

**Affiliations:** ^1^Psychiatry, Narcology and Medical Psychology; ^2^Ivano-FrankIskenderun National Medical University, Ivano-Frankivsk , Ukraine

## Abstract

**Introduction:**

Depression is a significant issue in chronic heart failure (HF), with a prevalence of about 20–40%, which is 4–5% higher than in the general population (Mbakwem A., et al., 2016).

**Objectives:**

The purpose of this study was to evaluate plasma fibronectin levels in patients with depression and chronic heart failure.

**Methods:**

A total of 80 patients with HF II-III NYHA classes due to chronic coronary artery diseases (CAD) were observed. All patients were divided into two groups: Group 1 - 20 individuals without signs of depression, and Group 2 - 60 individuals with depression. The diagnosis of HF was confirmed based on ESC guidelines (2021). Depression was diagnosed using several questionnaires (Zung Self-Rating Depression Scale, Beck Depression Inventory, Hamilton’s Depression Scale). Standard laboratory and instrumental tests were conducted. The plasma levels of fibronectin and interleukin-1β (IL-1β) were identified using ELISA methods. Statistical analyses were performed using Statistica system software, version 12.0.

**Results:**

The average plasma fibronectin concentration in patients with depression and HF was 1.24 times higher than a similar indicator in HF patients without depression: (259.63±5.71) μg/ml versus (203.41±9.51) μg/ml (p<0.05). The conducted correlation analysis indicated a moderate positive correlation between the level of fibronectin and the number of neutrophils in peripheral blood (r=0.35; p<0.05), the level of fibronectin and the magnitude of endogenous intoxication according to the erythrocyte absorption ability test (r=0.44; p<0.01), the level of fibronectin and IL-1β concentration (r=0.39; p<0.05), and an inverse correlation with left ventricle ejection fraction (r=0.32; p<0.05).

**Conclusions:**

Thus, the plasma fibronectin content in patients with depression and ischemic HF serves as a marker of the progression of myocardial remodeling processes and the intensity of the inflammatory process.

**Disclosure of Interest:**

None Declared

